# Regulatory mechanisms and clinical applications of tumor-driven exosomal circRNAs in cancers

**DOI:** 10.7150/ijms.82419

**Published:** 2023-05-08

**Authors:** Xiangyu Meng, Dong Yang, Bao Zhang, Yan Zhao, Zhichao Zheng, Tao Zhang

**Affiliations:** Department of Gastric Surgery, Cancer Hospital of China Medical University/Liaoning Cancer Hospital, Shenyang, Liaoning, China

**Keywords:** cancer, tumor-driven exosomes, exosomal circRNAs, mechanisms, clinical application

## Abstract

Malignant tumors seriously affect people's survival and prognosis. Exosomes, as vesicle structures widely existing in human tissues and body fluids, are involved in cell-to-cell transmission. Tumor-derived exosomes were secreted from tumors and involved in the development of carcinogenesis. Circular RNA (circRNA), a novel member of endogenous noncoding RNAs, is widespread in human and play a vital role in many physiological or pathological processes. Tumor-driven exosomal circRNAs are often involved in tumorigenesis and development including the proliferation, invasion, migration and chemo-or-radiotherapy sensitivity of tumor cell by multiple regulatory mechanisms. In this review, we will elaborate the roles and functions of tumor-driven exosomal circRNAs in cancers which may be used as potential cancer biomarkers and novel therapeutic targets.

## Introduction

Cancer is a major public health problem worldwide. GLOBOCAN 2020 suggested that there were 19,292,789 cancer cases and 9,958,133 cancer deaths globally, which indicated the burden of cancer incidence and mortality was rapidly growing worldwide 1. China is a country with a large population. Despite the rapid economic development and continuous improvement of people's living standards, the proportion of advanced tumors is high and the prognosis is poor 2. At present, it is believed that primary prevention is a particularly effective way to control cancer. In-depth exploration of the mechanism of cancer occurrence and development will provide more ideas and means for primary prevention of cancer 3.

In the early years, exosomes were then speculated to be a way for cells to excrete waste 4. However, with the deepening of research, the role of exosomes had been gradually paid more attentions to by researchers. Studies have shown that exosomes were involved in many biological processes including immune response, antigen presentation, cell differentiation, tumor growth and invasion 5. Exosomes were small membrane-bound extracellular vesicles with a diameter ranging from 40-160 nm, which could be detected in various bodily fluids and carry a wide repertoire of cellular components from their parental cells, including proteins, lipids, DNAs, mRNAs, and noncoding RNAs (ncRNAs) 6-8. Tumor-derived exosomes were secreted from tumors and involved in the development of tumor outgrowth. Exosomes from malignant cells could act on various recipient cells and eventually induced the growth, metastasis, angiogenesis, drug resistance or immune escape of adjacent tumor cells or normal recipient cells. Some tumor-derived exosomes had also been used as ideal diagnostic biomarkers and therapeutic targets for clinical application 9. More excitingly, using exosomes as naturally derived transport devices for the treatment in cancers is also being actively explored 8.

Circular RNAs (circRNAs), a class of endogenous noncoding RNA (ncRNA) that were produced by a non-canonical splicing event, were covalently closed, endogenous biomolecules in eukaryotes with tissue-specific and cell-specific expression patterns 10, 11. Their covalently closed ring structure made circRNAs have a high stability protecting these molecules from exonuclease-mediated degradation. Consistent with the fate of exosomes initially discovered which were regarded as ineffective products produced by metabolic processes in organisms, the biological functions of circRNAs in organisms had received little attention from researchers 12. In recent years, thousands of circRNAs in eukaryotes had been found with high-throughput RNA sequencing (RNA-seq) and circRNA-specific bioinformatics algorithms 13, 14. Shortly afterwards, many studies on circRNAs had been carried out. To date, by means of important non-coding functions including miRNA sponge and interacting with proteins, circRNAs had been implicated to participate in several human diseases, including cancers 15. Numerous studies have shown that circRNAs were often enriched in exosomes from cancers and played a regulatory role between tumor cells and tumor microenvironment (TME).

In this review, we will describe recent progress in our understanding of the regulatory mechanisms and biology functions of tumor-derived exosomal circRNAs which may provide more extensive content for the research and application of exosomal circRNAs in cancers.

## Tumor-driven exosomal circRNAs and malignant phenotypes

**Figure 1:**
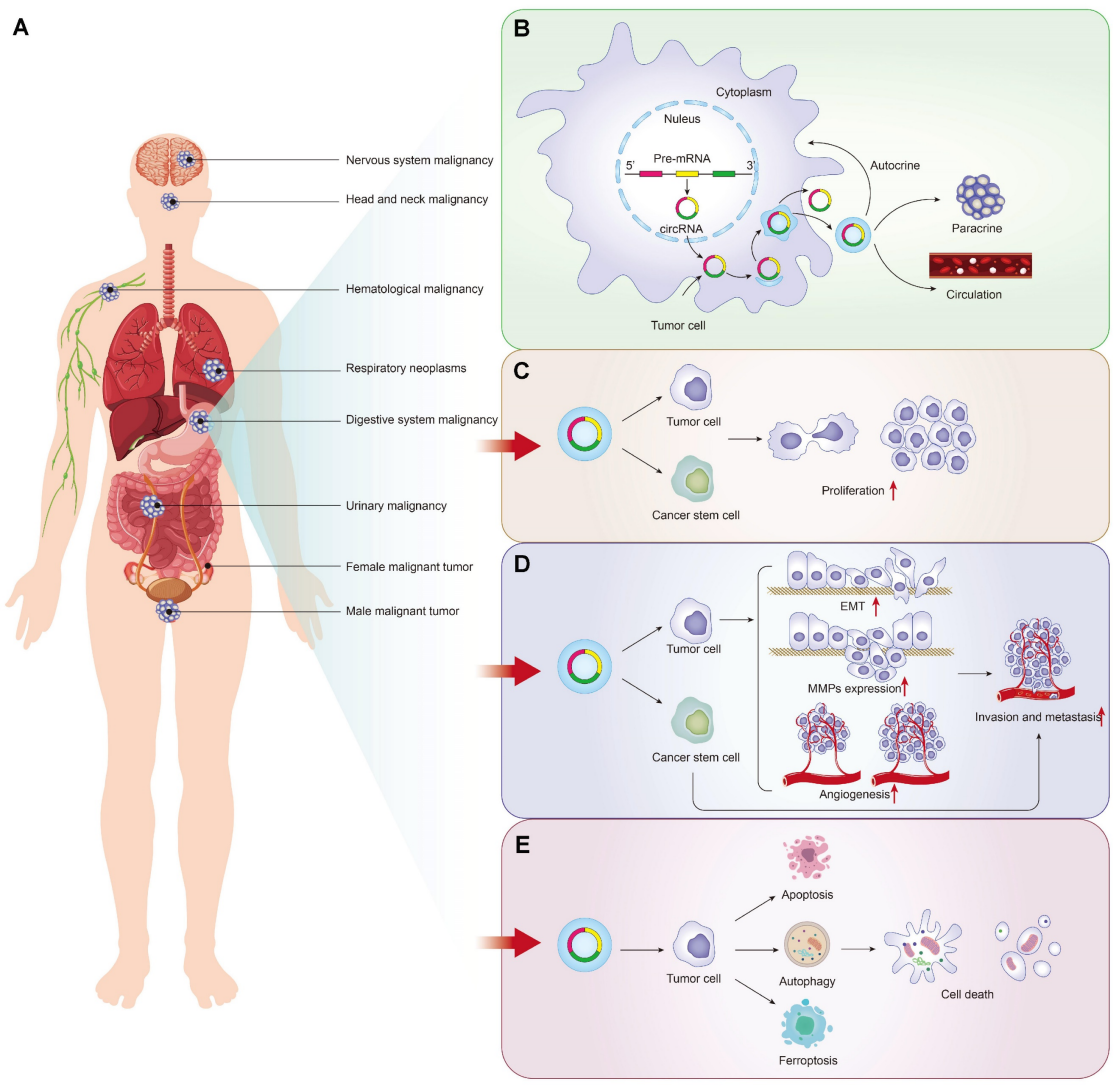
Tumor-driven exosomal circRNAs production and these association with malignant tumor phenotypes: **A**: Tumors drive exosome circRNAs associated malignancies; **B**: Production process and release pathway of tumor derived exosomal circRNAs; **C**: Tumor-driven exosomal circRNAs and proliferation in pan-carcinoma; **D**: Tumor-driven exosomal circRNAs and metastasis in pan-carcinoma; **E**: Tumor-driven exosomal circRNAs and apoptosis in pan-carcinomas.

Self-sufficiency in growth signals, insensitivity to growth-inhibitory (antigrowth) signals, evasion of programmed cell death (apoptosis), limitless replicative potential, sustained angiogenesis, and tissue invasion and metastasis were the six hallmarks of malignant cells 16. Uncontrolled proliferation, metastasis and programmed death of tumor cells play a vital role in cancer phenotypes. As shown in Figure 1A/1B, tumor-driven exosomal circRNAs are also involved in several malignant phenotypes described above. Detailed information of all tumor-driven exosome circRNAs retrieved in this review were shown in supplementary Table 1^17^-^125^.

### Tumor-driven exosomal circRNAs and proliferation

Independence on growth signaling is apparent in many cancers. Tumor-driven exosomal circRNAs make tumor cells to generate many of their own growth signals by inducing tumor cells to be blocked at G1/G0 phase. We observed the same phenomenon induced by tumor-driven exosomal circRNAs in many cancers. Cancer cells transfected with circRNAs exhibited the reduced cell viability and colony formation ability *in vitro*. *In vivo*,these tumor-driven exo-circRNAs also enhanced tumor volume and weight in nude mice (Table 1,^18^-^21^, ^25^-^29^, ^31^, ^33^, ^37^-^39^, ^41^, ^42^, ^44^-^46^, ^49^, ^50^, ^53^-^56^, ^60^-^64^, ^66^-^70^, ^75^, ^78^-^82^, ^84^-^86^, ^88^, ^90^, ^92^, ^93^, ^95^, ^97^-^99^, ^101^-^104^, ^106^, ^108^, ^111^, ^119^, ^120^, ^122^).

In addition to the effect of tumor-driven exosomal circRNAs on cell cycle, circRNAs can also regulate cyclin protein expression to regulate proliferation. For example, in HCC and esophageal cancer, exosomal circRNA_100284^33^ and circ-SFMBT2^67^ regulated the cell cycle via up-regulating cyclin-D1 and increased proliferation and oncogenic capacity. Ki67 is a nuclear antigen closely related to tumor cell proliferation and its expression is mainly concentrated in M phase of cell cycle 126. The abnormal expression of some tumor-driven exosomal circRNAs directly reflects the proliferation rate and proliferation activity of malignant tumor cells. In NLCSC, exo-circSHKBP1 induced cell proliferation by increasing the Ki67 protein levels 88.

Some circRNA-related special mechanisms that regulated tumor cell proliferation were also identified in the literature searching. Cancer stem-like cells were defined as a small population of tumor cells which were able to self-renew and generate the neoplasm by unlimitedly proliferating 127. In colon cancer, breast cancer and malignant pleural mesothelioma cancer, circ-ABCC1^36^, circHIF1A 99 and circPLK1^92^ induced cancer cell proliferation by promoting cancer cell stemness. In addition, cancer stemness-related genes such as SOX2, ABCG2, NANOG, and OCT4 were also regulated by tumor-driven exosome-derived circRNAs, for example, exo-circSHKBP1 in NLCSC 87 (Figure 1C).

### Tumor-driven exosomal circRNAs and metastasis

The distant metastases of tumor cells are the cause of 90% of human cancer deaths 128. Many biological processes, such as tumor cell movement, adhesion and extracellular matrix degradation, are closely related to tumor metastasis 129. Growing evidences indicate that tumor-driven exosomal circRNAs were involved in the regulation of the vital process. We found that tumor-driven exosomal circRNAs in many cancers, as oncogenes or tumor suppressor genes, can regulate the invasion and metastasis of tumor cells both *in vivo* and *in vitro* (as shown in Figure 1D). Mechanistically, most included studies indicated that most tumor-driven exosomal circRNAs mediate tumor cell invasion and metastasis by forming competing endogenous RNAs (ceRNAs) regulatory axis with miRNA and mRNA (as shown in Table 1). It is well known that circRNAs can act as miRNAs sponges to adsorb them, thereby weakening the inhibition of downstream mRNA transcription induced by miRNAs 10. Changes in the physical coupling of cells to their microenvironment and activation of extracellular proteases were the two cores of tumor cell metastasis 16. Several classes of proteins involved in the process including cell-cell adhesion molecules, integrins and extracellular proteases 130, 131. The epithelial-to-mesenchymal transition (EMT) is a cell-biological program that induced normal epithelial cell to mesenchymal traits, thus finally enabling carcinoma cells to complete many of the steps of the invasion-metastasis cascade 132. The deregulation of adhesion-related proteins plays an important role in EMT of cancers. In many cancers, tumor-driven exosomal circRNAs induced the tumor metastasis by regulating the expression of EMT-related protein makers. These tumor-driven exosomal circRNAs include circFNDC3B 38, circCOG2 50, circ_100395 79, circFBXW8 82, circFARSA 89, circ_0001142 103, circ-PDE8A 69, and circ_0020256 64. In addition to regulating the expression of EMT-related proteins, tumor-driven exosomal circRNAs also affected changes in other adhesins to promote tumor metastasis. In gastric cancer, circ_0000260 promoted the ability of migration and invasion in CDDP resistant GC cell by inducing an increase of the expression of fibronectin and vitronectin 61. In HCC, circ-0004277 overexpression significantly promoted the metastasis of HCC cell by inhibiting the expression of ZO-1 (a member of the membrane-associated guanylate kinase (MAGUK) family of proteins, and regulates adherens junctions) 17.

Extracellular proteases were very important for activation of matrix metalloproteinase (MMP). MMPs were significantly involved in metastasis. In HCC, exo-circ-0072088 could regulate the expression of MMP16 and promote the invasion and migration of HCC cells 28. Similar mechanisms have been observed for GC 55 and esophageal cancer (EC) 67.

The oxygen and nutrients supplied by the vasculature are crucial for tumor cell function and survival. Lots of positive signals encourage angiogenesis in many cancers. Exo-circRNAs also promoted or inhibited the metastasis of tumor cells by regulating soluble mediating cell-matrix and cell-cell association-related factors and their receptors. In CRC, exosomal circFNDC3B, as a tumor suppressor, decreased the generation of the angiogenic activator VEGFA to inhibit the angiogenesis 36. On the contrary, circ_0007334 promoted the migration and invasion of CRC cells by restraining the VEGFA expression of HUVECs *in vivo* and *vitro* 53.

Some special regulatory mechanisms still need to be emphasized. For example,in CRC, exo-circ-ABCC1 promotes the CRC cell migration by regulating CRC cell stemness^36^. circRHOBTB3 regulate intracellular reactive oxygen species (ROS) levels to inhibit tumor cell EMT by interacting with metabolic enzymes such as ENO1 and ENO2^52^.

### Tumor-driven exosomal circRNAs and apoptosis

Apoptosis is programmed cell death characterized by an elaborate sequence of morphological events such as nuclear condensation (pyknosis) and fragmentation (karyorrhexis), along with blebbing of the plasma membrane 133. Apoptosis is needed to maintain the normal turnover of cells and embryonic development by facilitating cell death induced by a variety of stimuli. Any alteration to the normal apoptosis pathway (including the intrinsic and extrinsic pathways) can lead to various diseases including cancers 134. Large studies indicated that apoptosis defects in cancers were closely related to the cause of chemoresistance 135, cancer metastasis 136, cancer angiogenesis 137, TME 138, tumor-promoting inflammation 139, tumor immune infiltration 140 and cancer stem cells 141. In addition to the key molecular components of the intrinsic or extrinsic apoptotic pathway involved in the management of apoptosis, circRNAs also played an important role in this process. As presented in Table 1, we found by reviewing that many tumor-driven exosomal circRNAs indeed to be involved in cancer progression by promoting or inhibiting the apoptosis process.

Autophagy, a special programmed cell death pathway, can be defined as a cellular process meant for the degradation and elimination of misfolded proteins and damaged organelles that function in adaptation to cellular stress events like starvation, development, cell death, and tumor suppression 142. Studies had shown that the interference of autophagy in the process of apoptosis results in failure to achieve cell death 143. Autophagy facilitates the cell survival mechanism through which the cells adapt to stress conditions and survive with stress, escaping cell death by apoptosis 143. We also observed that tumor-driven exosomal circRNAs played an important role in autophagy process. For instance, circ-PVT1 knockdown repressed CDDP resistance via inhibiting autophagy in GC cells 56. In breast cancer (BC), circ_0001142 could induce M2 polarization by regulating miR-361-3p and then affecting the level of autophagy in BC cells 103. As another special programmed cell death pathway, ferroptosis also attracted more and more attentions from researchers. It is a newly reported iron-dependent PCD process that is characterized by the accumulation of an iron-dependent lethal lipid ROS response 144. It is worth noting that some exosomal circRNAs could regulate programmed cell death in cancer via inducing ferroptosis. In lung adenocarcinoma (LUAD), exosomal circRNA_101093 from LAC cells upregulated expression of FABP3 protein and desensitize LUAD cells to ferroptosis via a FABP3-dependent reduction 77.

It can be concluded that tumor-driven exosomal circRNAs play vital roles in proliferation, metastasis, and apoptosis of cancers. This provides a general direction for the subsequent exploration of the mechanism in the genesis of various cancers (Figure 1E).

## Roles and mechanisms of tumor-driven exosomal circRNAs in carcinogenesis

With the continuous development of next-generation sequencing approaches in the past two decades, the level of research on the molecular mechanisms of tumorigenesis has changed and became more systematic, which revealed that the presence of numerous ncRNAs enriches the molecular mechanisms of carcinogenesis 145. As a new functional player in cancer biology, tumor-driven exosomal circRNAs have emerged as the important roles in regulating the development of cancers via different molecular mechanisms (including binding to proteins,sponging miRNAs, and interfering with the splicing of other RNAs) by means of the membrane structure of exosomes protecting circRNAs from degradation by enzymes and other chemicals.

### ceRNA network with miRNA and mRNA

CeRNAs hypothesis was first put forward by Salmena et al. in 2011^146^. Among the many circRNAs regulatory mechanisms, circRNA/miRNA/mRNA network regulation was one of the most influential and deregulated mechanisms in cancers. Serving as miRNA sponge, circRNAs with miRNA binding sites bond directly to the corresponding one or more miRNAs to inhibit miRNA activity and thus regulated the expression of target genes 147. Overwhelming evidence according to the results shown in Table 1 had demonstrated the profound impact of exosomal circRNA-mediated ceRNA interactions on multiple processes and events in the pathogenesis of diverse cancers such as cell proliferation, invasion, migration, apoptosis, chemoresistance or tumor microenvironment. Importantly, the roles of circRNAs in these processes were completed by exosome encapsulation and delivery. Thus, the regulatory mechanism of ceRNA network centered on exosomal circRNA played an important role in the management of biological behavior of malignant tumors.

### Tumor-driven exosomal circRNAs regulate downstream gene expression

#### CircRNAs-protein interaction regulation pathway

**Figure 2:**
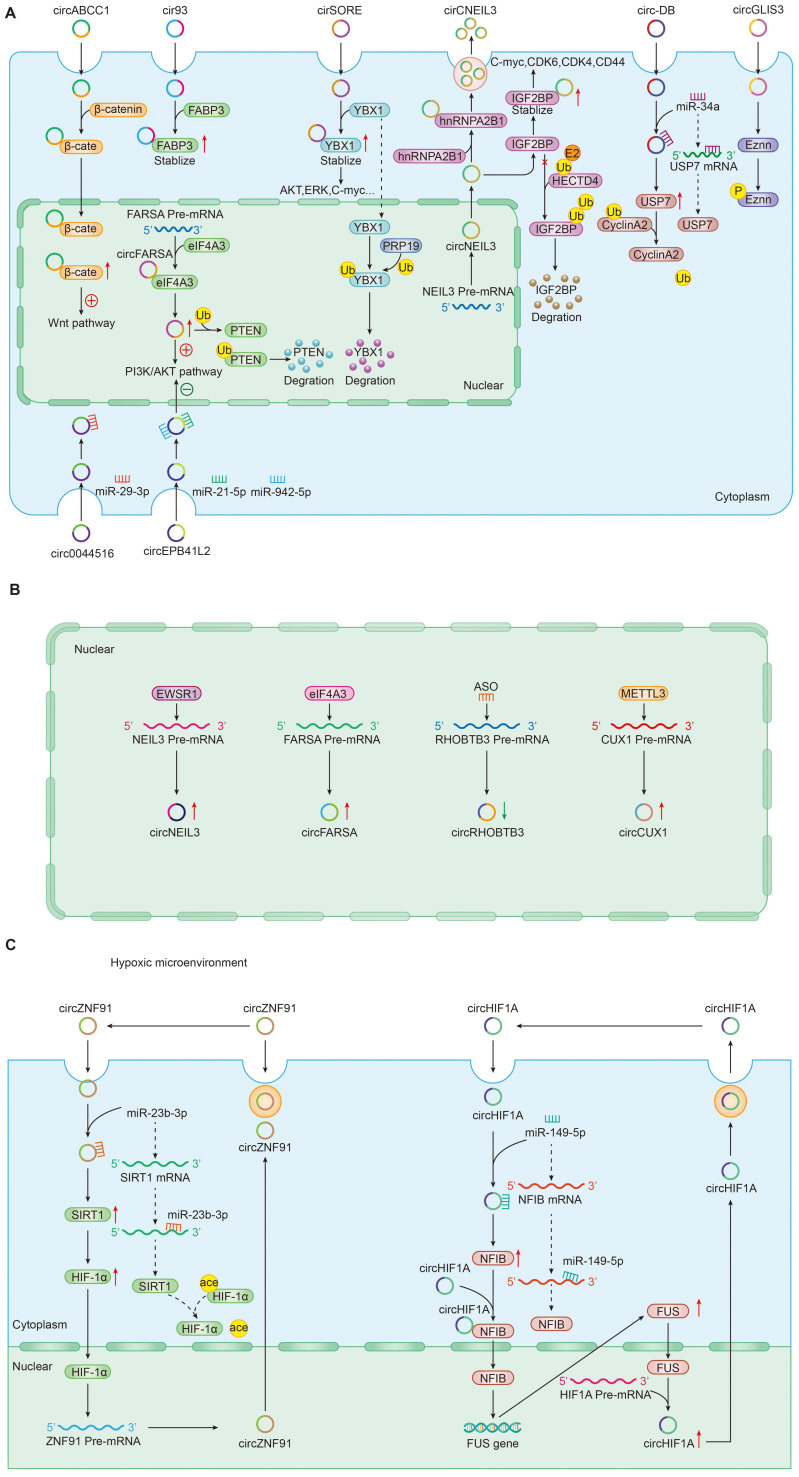
Roles and mechanisms of tumor-driven exosomal circRNAs in carcinogenesis: **A**: Tumor-driven exosomal circRNAs regulate downstream gene expression; **B**: Upstream factors regulate tumor-driven exosomal circRNA expression; **C**: Feedback-loop regulating mechanism by tumor-driven exosomal circRNAs.

In addition to the most well-accepted function of ceRNAs as miRNA sponges, tumor-driven exosomal circRNAs also directly interacted with proteins to perform physiological functions. Recent studies had shown that circRNA-protein interactions may play critical roles in a variety of diseases 20, 22, 35, 78, 92, 117; Cytoplasm-retained circRNAs usually perform their functions by acting as scaffolds for RNA-binding proteins or other proteins 15, 148 (Figure 2A). For instance, circ-ABCC1 in CD133+cell extracted from CRC cells upregulated the expression of β-catenin in the nucleus and inhibited the expression of β-catenin in the cytoplasm by binding with β-catenin, which activating the wnt pathway, then mediated CRC cell stemness and metastasis 36. In LUAD, exosomal cir93 increased the level of intracellular cir93 and interacted with FABP3, thus further desensitizing LUAD cells to ferroptosis 77. In NSCLC, by help of the cyclization induced by eIF4A3, exosomal circFARSA promoted macrophage polarization to the M2 phenotype by downregulating PTEN expression and activating the PI3K/AKT pathway 89. In HCC, circRNA-SORE from the cytoplasm binds YBX1 via the Y box sequence and prevent YBX1 from translocating into the nucleus to stabilize YBX1, which and then decreasing the ubiquitination of YBX1 induced by PRP19 in nucleus. Finally, the downstream target gene expressions of YBX1 including AKT, Raf1, ERK, c-Myc, and TGF-β1 would be affected and the patients treated with sorafenib developed resistance 22. In glioma, circNEIL3 interacts with IGF2BP to inhibit the ubiquitin/proteasome-mediated degradation of IGF2BP3 to promote its protein expression and as well as that of its downstream targets, including CDK4/6, CD44 and c-MYC, thereby promoting malignant progression of glioma 73.

#### CircRNAs - miRNA interaction regulation pathway

In two articles, we also found a circRNA-miRNA interaction mode independent of the circRNA-centered ceRNA network regulation mechanism, which also played an important role in regulating cancer progression (Figure 2A). For example, Li et al. determined that exosomal circ_0044516 downregulate miR-29-3p expression by interacting with miR-29-3p to promote the proliferation and metastasis of prostate cancer cells 123. In CRC, Jiang et al. found that exosomal circEPB41L2 sponged miR-21-5p and miR-942-5p to repress colorectal cancer progression by regulating the PTEN/AKT signalling pathway 47. It was worth noting that the pattern in which this circRNA-miRNA interaction affects the activation of downstream PTEN/AKT signalling pathway identified by Jiang et al. does not be identified for specific mRNA targets. In essence, circRNA still plays the role of miRNA sponge in circRNA-miRNA interaction mechanism.

#### CircRNAs and epigenetic regulation

Epigenetics is defined as a heritable change in gene expression or chromosomal stability by utilizing DNA methylation, histone covalent modification or non-coding RNAs without a change in DNA sequence 149. These effects of epigenetic changes had been well documented to contribute to tumor progression 150. As a vital epigenetic mark, ncRNAs including lncRNA, circRNA, and miRNA mis-regulation eventually leaded to the activation of oncogenes or the deactivation of tumor suppressor genes governing the hallmarks of cancer such as sustaining proliferative signaling, evading growth suppressors, resisting cell death, enabling replicative immortality, inducing angiogenesis, and activating invasion and metastasis 151. In this review, we also found that many tumor-driven exosomal circRNAs regulated oncogene or tumor suppressor gene expression by participating in or managing epigenetic alterations (Figure 2A). Firstly, these circRNAs could regulate protein ubiquitination and degradation at the post-transcriptional level. In gliomas, Pan et al. found that circNEIL3 interacts with IGF2BP in the cytoplasm, enhancing the stability of IGF2BP3 protein and inhibiting the E3 ubiquitin ligase HECTD4-mediated degradation of IGF2BP3 at the post-transcriptional level to promote the polarization of macrophage toward an immunosuppressive phenotype enabling gliomas to acquire angiogenic and immunosuppressive properties in turn promoting tumor progression. Meanwhile, exosomes wrapped circNEIL3 and secreted it to surrounding immune cells, making them lose their immune regulatory function and leading to carcinogenesis 73. In HCC, exosome circ-DB and circRNA-SORE were also involved in the regulation of protein ubiquitination. Exosomal circ-DB absorbed miR-34a and enhanced the deubiquitination of cyclin A2 mediated by USP7, promoting HCC growth 21. Exosomal circRNA-SORE stabilized YBX1 in the cytoplasm, thus preventing YBX1 from subsequently be degraded by the E3 ligase PRP19 in the nucleus and enhancing the sorafenib resistance of HCC 22. In NSCLC, exosomal circFARSA regulated PTEN expression at a post-transcriptional level and activated the PI3K/AKT signaling pathway in macrophages by promoting PTEN ubiquitination and degradation, which induced M2 polarization 89. Besides ubiquitination mediated by, circRNAs could induce protein phosphorylation in cancers. For example, exosomal circGLIS3 directly binds with p-Ezrin (T567) and elevates p-Ezrin (T567) level at the post-transcription, promoting the Ezrin T567 phosphorylation and contributing to the invasion and angiogenesis of glioma 74.

### Upstream factors regulate tumor-driven exosomal circRNA expression

RNA-binding proteins (RBPs) were the most common factors regulating the expression of circRNAs in upstream. Large bioactivities such as tumor cell proliferation, differentiation, and apoptosis were closely related to the RBPs 152. Studies suggested that various RBPs induced the upregulation of tumour-associated circRNAs^10^, ^105^. In glioma, an RNA-binding protein EWSR1 could bind to sequence 1 and sequence 3 of NEIL3 pre-mRNA and the binding ability was significantly upregulated under hypoxic conditions 73. The study from Chen et al. indicated that eIF4A3 could directly bind to the sequences flanking circFARSA to mediate its circularization and expression 89.

In addition to RBPs that control circRNA expression in upstream, some special factors were also involved in regulating circRNA expression. In CRC, for example, Chen et al. found that antisense oligonucleotides artificially synthesized could negatively regulates circRHOBTB3 circularization and expression, inhibiting CRC growth and metastasis *in vitro* and *in vivo* 52. In hypopharyngeal carcinoma, METTL3, a predominant methyltransferase for m6A modifification, could bind to circCUX1 and stabilize the expression of circCUX1 through m6A methylation modification regulating the progression of radiotherapy tolerance 109. The above mechanisms were shown in Figure 2B.

### Feedback-loop regulating mechanism

Numerous studies had associated feedback loop regulation with the development and therapeutic response of cancers 153. The complex and variable signal networks led to the difficulties for cancers to develop precise targeted therapy drugs.

The feedback loop usually consists of a negative feedback loop and a positive feedback loop inhibiting or activating upstream and downstream signaling proteins 154. In the process of exploring the mechanism of tumor-driven exosomal circRNAs mediated tumorigenesis, we also found the existence of feedback loop regulation mechanism (Figure 2C). In pancreatic cancer, hypoxic exosomal circZNF91/miR-23b-3p/SIRT1/HIF-1a formed a positive feedback loop that co-regulates gemcitabine resistance in pancreatic cancer cells under hypoxia. In this positive feedback loop, circZNF91 upregulated SIRT1 expression via competitively binding to miR-23b-3p and SIRT1 stabilizes HIF-1α protein via decreasing the acetylation of HIF-1α in pancreatic carcinoma (PC) cells,thus increasing the protein level of HIF-1α. As a transcription factor, HIF-1α could bind to the region at upstream of circZNF91 transcription start sites and promoted endogenous transcription of circZNF91^68^. Another a positive feedback loop was composed of circRNA 0001445/miRNA-127-5p/SNX5 involving in tumor progression including proliferation, migration and invasion. Similar feedback loops had been also found in triple-negative breast cancer. Exosomal circHIF1A could act as a miR-149-5p sponge and increase nuclear factor IB (NFIB) expression via post transcriptional regulation. In addition, circHIF1α could also bind to the NFIB protein directly in the cytoplasm and promote NFIB nuclear translocation. Interestingly, NFIB could act as a transcriptional activator of FUS (the FET family of ubiquitously expressed RNA-binding proteins) and upregulate the protein level of FUS. Meanwhile, in the 5' end flanking its intron, circHIF1A could bind to FUS as a modulator of circHIF1A formation. Finally, a positive feedback loop was formed by the circHIF1A/NFIB/FUS axis, thus increasing the TNBC growth and migration 105.

## Tumor-driven exosomal circRNAs and metabolism

**Figure 3:**
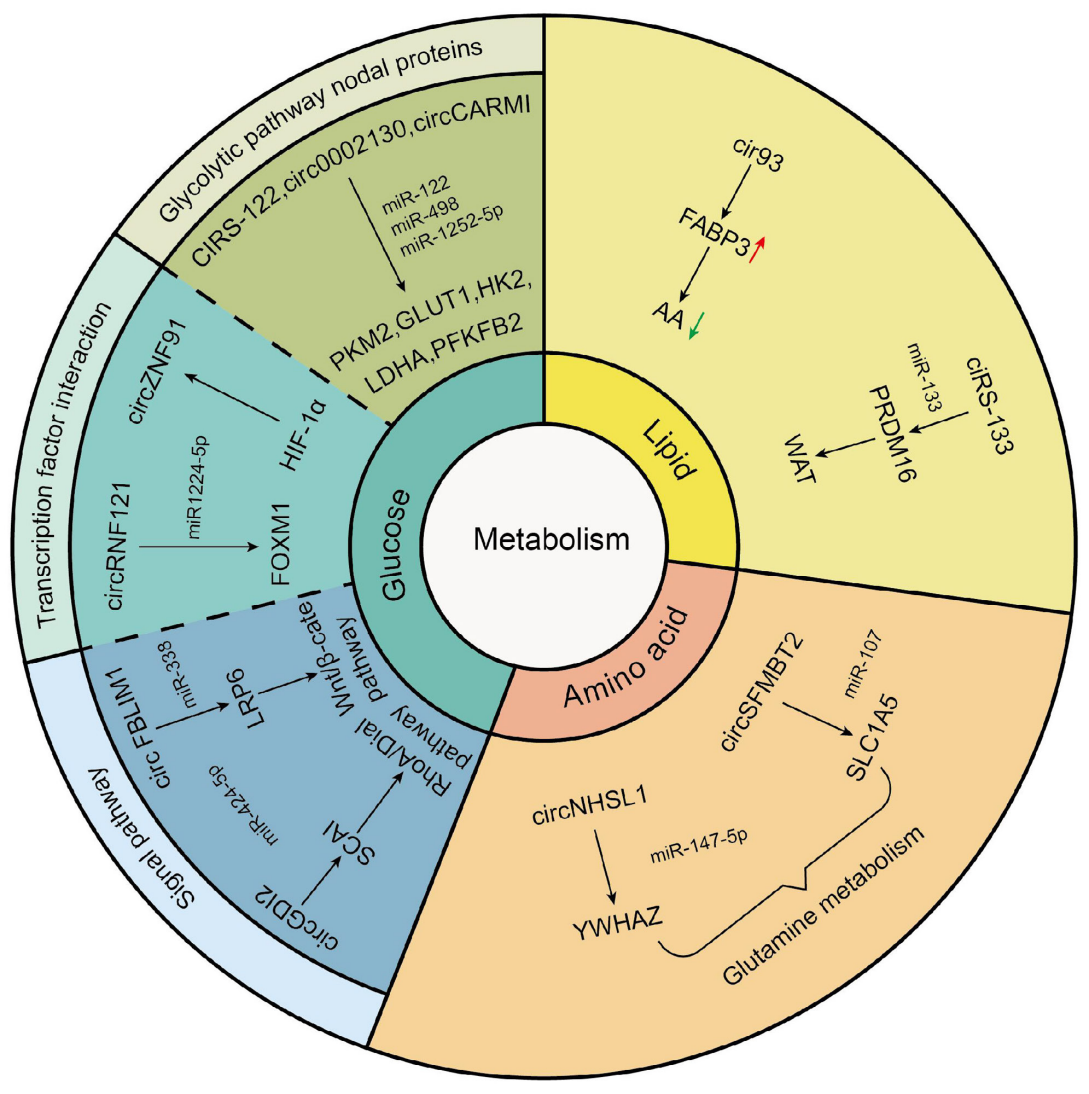
Tumor-driven exosomal circRNAs and metabolism

Cancer has long been considered a genetic disease characterized by a myriad of mutations that drive cancer progression 16. But why do mutations in very different set of genes from most genetic disorders including cancers yield the same phenotypic outcome?^155^ Recent accumulating evidence indicated that the dysregulated metabolism in cancer cells was more than a hallmark of cancer but might be the underlying cause of the tumor 155. Since the notion of “Warburg effect” was proposed by Otto Warburg in 1927, cancers have been gradually identified as a metabolic disease 156. Mechanically, these genetic alterations create an altered metabolic state allowing cancer cells to generate the large quantities of macromolecules (amino acids, nucleotides, and fatty acids) and metabolic intermediates which were required to fuel rapid cell growth and division resulting in carcinogenesis 157. As an emerging category of regulatory molecules, circRNAs had been verified to control cancer metabolism 158. In this review, we also found that tumor drives exosomal circRNAs participated in metabolic regulation (Figure 3).

### Tumor-driven exosomal circRNAs and glucose metabolism

In glucose metabolism, the Warburg effect is instrumental in malignancy. Normally, cells consume glucose to generate ATP through oxidative phosphorylation (OXPHOS) under the aerobic condition and channel into glycolysis only under the anoxic condition. However, cancer cells prefer glycolysis even in the normoxic 159. On the one hand, this metabolism method relieved oxidative stress injury caused by mitochondria. One the other hand, excess lactic acid produced by the Warburg effect helped avoid immune surveillance environment 160, 161. Tumor-driven exosomal circRNAs participated in glycolysis just by regulating expression of glucose transporters, metabolic enzymes, and oncogenes. The glucose transporters (GLUTs) and metabolic enzymes (including hexokinase (HK), 6-phosphfructa-1-kinase (PFK), lactate dehydrogenase, and pyruvate kinase) played a vital role in glycolysis progress. For instance, in CRC, exosomes derived from chemoresistant CRC cells could transfer ciRS-122 across cells and promote glycolysis to reduce drug susceptibility through the miR-122-PKM2 axis in CRC 41. In NSCLC, exosomal hsa_circ_0002130 targeted miR-498 to regulate GLUT1, HK2 and LDHA and induce glycolysis, which enhanced osimertinib resistance 86. A similar mechanism has been found in BC. BC stem cell exosome-derived circCARM1 played an important role in breast cancer cell glycolysis by sponging miR-1252-5p which regulated PFKFB2 expression 104.

Besides regulating expression of glucose transporters and metabolic enzymes, exosomal circRNAs also facilitated glycolysis by affecting functions of transcription factors (TFs). For example, Jiang et al. found that exosomal circ-RNF121 modulated CRC malignant progression via sponging miR-1224-5p to regulate the expression of Forkhead box M1 (FOXM1), an important transcription factor 46. In addition, in pancreatic cancer (PCA), HIF-1α could bind to the region at upstream of circZNF91 transcription start sites and promoted endogenous transcription of circZNF91, thus regulating HIF-1α-associated glycolysis and increased GEM resistance in normoxic PCA cells 68.

Apart from the above influencing mechanisms, tumor-driven exosomal circRNAs also participated in glycolysis by affecting other signaling pathways. For instance, exosomal circGDI2 acted as a miRNA sponge through targeting the miR-424-5p/SCAI axis to regulate the RhoA-Dia1 signal transduction pathway weakening oral squamous cell carcinoma (OSCC) cell glycolysis 106. In HCC, exosomal circFBLIM1 increased LRP6 expression by sponging miR-338 to regulate the activation of Wnt/b-catenin pathway and induce glycolysis and progression of patients with HCC 29.

No matter what mechanism circRNAs participated in regulating glycolysis, its core function was to act as a miRNA sponge to regulate the expression of downstream target genes. Thus, this ceRNA regulation played an important role in the management of glycolysis.

### Tumor-driven exosomal circRNAs and lipid metabolism

Abundant lipid production is essential for cancer cell growth in tumor metabolism which needed adequate fatty acids (FAs) to replicate cellular membranes thus ensuring unlimited proliferation of cancer cells. Endogenous fatty acid synthesis (FAS) is triggered in various tumors 162. In this review, we also found that tumor-driven exosomal circRNAs were involved in lipid metabolism in tumor cells. For example, arachidonic acid (AA) was a poly-unsaturated fatty acid and critical for ferroptosis associated increased peroxidation in the plasma membrane. Exosome released from LUAD cells increased intracellular cir93 to upregulate FABP3 and desensitize LUAD cells to ferroptosis via a FABP3-dependent reduction in global AA and prevention of AA incorporation into the plasma membrane 77.

In addition to participating in fatty acid metabolism, tumor-driven exosomal circRNAs played a vital role in adipose tissue transformation. It was well known that adipose tissue includes white adipose tissue (WAT) and brown adipose tissue (BAT). The former was used to store energy for the body and the latter was used for producing heat against cold and obesity conditions 163. Although brown adipocytes can be induced within WAT depots, called as WAT browning, high percentage of WAT browning in adipose tissue was closely related to poor prognosis and death in patients 164, 165. Thus, WAT browning in adipose tissue was undesired among tumor patients. The study of Zhang et al. indicated that exosomes derived from GC cells delivered ciRS-133 into pre-adipocytes, promoting the differentiation of pre-adipocytes into brown-like cells by activating PRDM16 and suppressing miR-133, which involved in WAT browning and played a key role in cancer-associated cachexia 54.

### Tumor-driven exosomal circRNAs and amino acid metabolism

Glutamine, a non-essential amino acid with an amine functional group, is the most abundant amino acid circulating in the bloodstream. Glutamine was a major source of energy apart from glucose in proliferating cancer cells. The hydrolysis of glutamine also was a metabolic route essential for cancer cell survival and growth 166. We found that some studies had investigated the impact of exosomal circRNAs on glutamine metabolism. For example, in gastric cancer, exosomal circNHSL1 could affect YWHAZ gene expression by sponging miR-149-5p and induce the glutaminolysis of GC cells 63. In esophageal cancer, Chang et al. indicated that exosomal circ-SFMBT2 increased glutamine metabolism in EC cells by targeting miR-107 and upregulating SLC1A5 expression to promote the malignant development of EC 67.

## Tumor-driven exosomal circRNAs and clinical applications

### Tumor-driven exosomal circRNAs and diagnosis

The clinical application of tumor-driven exosomal circRNAs had rapidly attracted more and more attention. With the continuous development of high-throughput sequencing technology and the convenience of extracting exosomes from patient serums, a large number of studies had pointed out that many tumor-driven exosomal circRNAs could be used as molecular markers of some tumors to support diagnosis (Table 2). In CRC, for example, the combination of exosomal circLPAR1, CEA and CA19-9 increased the sensitivity and specifcity of diagnosis in CRC patients 35. Circulating exosomal hsa-circ-0004771 could differentiate CRC patients from healthy controls 43. In LUAD, serum exosomal circRNA-002178^84^ and hsa_circ_0069313^83^ could serve as a novel diagnosis biomarker for LUAD. Similarly, serum exosomal circKIAA1244 was also proved to serve as a novel circulating biomarker for detection of GC from healthy controls 57.

The detection and localization of tumors at their early stage is extremely crucial for treatment of cancer, and then to markedly prolong the life of the patients. Exosomal circRNAs were also being recognized as vital screening factors of early cancers. In CRC, a study suggested that the detection of early-stage CRC was confirmed by the detecting of serum exosomal circ-PNN 40. In GC, Shao et al. found by bioinformatics analysis that hsa_circ_0065149 in plasma exosomes had higher sensitivity and specificity than traditional clinical biomarkers such as CEA, CA199, and CA125 and could be used as an indicator for early GC screening and prognosis prediction 58. In addition, there had been many reports on the combination of multiple exosomal circRNAs to detect early cancer. For instance, the study from Xia et al. suggested that the exosome derived hsa_circ_0055202, hsa_circ_0074920 and hsa_circ_0043722 were firstly found to be used as the potential biomarker for early screening for glioblastoma multiforme (GBM) in healthy populations 71. In astrocytoma, the features of exosomal circRNAs including hsa_circ_0003828, hsa_circ_0075828, and hsa_circ_0002976 from primary high-grade astrocytoma cells and cell-derived exosomes could be also used for screening in early high-grade astrocytoma 76.

Meanwhile, some tumor-driven exosomal circRNAs were proved to play a vital role in predicting cancer recurrence or metastasis and indicating poor prognosis. **I)** Predicting recurrence: The study from Wang et al. indicated that a risk score model consisted of eight circRNAs was used to predict the biochemical recurrence of prostate cancer (PCa) patients 121. In OSCC, high exosomal circ_0000199 expression indicated the high tumor recurrence rate and mortality rate 107. Likewise, high exosomal circMYC expression in the multiple myeloma patients' serums implied that the patients might have higher relapse rates and higher mortality rates 116. In laryngeal squamous cell carcinoma, three serum exosomal circRNAs consisted of circ_0019201, circ_0011773 and circ_0122790 could be used as an early prediction model for occurrence of tumor 112. **II)** Poor prognosis prediction: Through the detection of serum exosomes, the abnormal expression of some tumor-driven exosomal circRNAs also indicated the poor prognosis of cancer patients. For example, four exosomal circRNAs including hsa_circ_0005019, hsa_circ_0000880, hsa_circ_0051680, and hsa_circ_0006365 were used as markers for monitoring high-grade astrocytoma prognosis 76. High exo_circRNA_0056616 expression represented a potential biomarker for screening and identifying lymph node metastasis risk in LUAD. **III)** Prediction treatment effect: In addition, serum exosomal circRNAs had also been used by researchers in the prediction of therapeutic efficacy. In nasopharyngeal carcinoma, the expression status of exosomal circMYC could be used as a biomarker for differentiating radioresistant patients from radiosensitive patients with nasopharyngeal carcinoma 110. It can be seen that the detection of serum exosomal circRNAs has become an important means and method for doctors in cancer diagnosis, early screening, poor prognosis prediction and treatment expectation.

### Tumor-driven exosomal circRNAs and drug-therapy or radiotherapy sensitivity and resistance

Drug-therapy and radiotherapy played an important role in the treatment of most solid cancers. Drug-therapy or radiotherapy resistance was a critical factor for tumor treatment failure and the development of locoregional relapse and distant metastases which seriously affected the prognosis of tumor patients. Different from the primary drug or radiotherapy resistance caused by genetic change, this acquired resistance were often induced by regularly exposing tumor cells to conventional drug-therapy or radiotherapy 167, 168. It followed that the prediction and overcoming of drug-therapy or radiotherapy resistance were particularly important. As a novel kind of ncRNA, circRNAs had been shown to be involved in the induction of drug-radiotherapy resistance 169, 170. Particularly, tumor-driven exosomal circRNAs were encapsulated and released by exosomes to change nearby cell characteristics in the tumor microenvironment (Table 2).

#### Tumor-driven exosomal circRNAs and drug-therapy

Tumor-driven exosomal circRNAs played an important role in cancer drug therapy. In terms of chemotherapy, in this review, we found that many tumor-driven exosomal circRNAs were involved in the mechanism of chemotherapy resistance of several tumors. In CRC, circ_0006174 in exosomes from doxorubicin-resistant CRC cells spread doxorubici resistance among CRC cells via the miR-1205-mediated overexpression of CCND2^37^. Exosomes derived from chemoresistant CRC cells could transfer ciRS-122 across cells and promote cell glycolysis to reduce drug susceptibility in chemosensitive cells through the miR-122-PKM2 axis 41. Differentially upregulated hsa_circ_0000338 in exosomes could serve as a potential biomarker for early prediction of chemoresistance in CRC 48. In LUAD, circ_0076305 promoted CDDP resistance of NLCSC cells through the miR-186-5p-ABCC1 axis 81. Exosomal circ_PIP5K1A inhibited CDDP sensitivity by regulating miR-101/ABCC1 axis in NLCSC 85. In PCA, exosomal circZNF91 was critical for the hypoxic exosome-promoted GEM resistance in normoxic cancer cells 68. In glioma, hsa_circ_0042003 in exosomes derived from temozolomide (TMZ) resistance U251 cells conferred the resistance of U251 cells to TMZ 72. In bone tumors, exosomal hsa_circ_103801 reduced the sensitivity of osteosarcoma cells to CDDP and strengthened the promotive effect of exosomes on the chemoresistance of osteosarcoma cells CDDP 124. In addition, the expression of circMYC in circulating exosomes also played a vital role in bortezomib-resistant patients with multiple myeloma (MM) 116. In ovarian cancer (OC), Luo et al. found that circFoxp1 conferred CDDP resistance to epithelial OC cells via sponges of miR-22 and miR-150-3p positively regulating the expressions of CEBPG and FMNL3 gene.

In addition to the regulatory role of chemotherapy resistance in cancers, tumor-driven exosomal circRNAs also played an important role in the resistance of targeted therapy and immunotherapy. For example, the study from Zhong et al. indicated that hsa_circ_0058493 was significantly correlated with imatinib-resistance in CML 117. In HCC, exosomal circRNA-SORE was critical for maintaining sorafenib resistance 22. In LUAD, tumor-derived exosomal circRNA_102481^80^ and hsa_circ_0002130^86^ both contributed to osimertinib resistance via ceRNA regulation pathway. Meanwhile, some exosomal circRNAs were also reported to be involved in immunotherapy resistance. In HCC, for example, exosomal circUHRF1 could enhance HCC resistance to anti-PD1 therapy by upregulating TIM-3 expression and suppressing miR-449c-5p activity 19. Lu et al. found that high exosomal circTMEM181 favored the immunosuppressive microenvironment and endowed anti-PD1 resistance via sponging miR-488-3p and upregulating CD39 expression in macrophages 27. In LUAD, exosomal circUSP7 was found to induce CD8+ T cell dysfunction and anti-PD1 resistance by regulating the miR-934/SHP2 axis in NSCLC 90. Surprisingly, we also found that some tumor-driven exosomal circRNAs, such as Cdr1as 94 and hsa-circRNA-G004213^26^, could increase the sensitivity of drug therapy and improve the effect of tumor treatment in several cancers. These results demonstrated that exosomal circRNAs played multiple roles in regulating drug treatment mechanisms (Table 2).

#### Tumor-driven exosomal circRNAs and radiotherapy

Radiation therapy (RT) is the most effective method of cytotoxic treatment based on ionizing radiation, which was used in the treatment of most solid cancers 171. However, the high frequency of relapses after RT was still found in different cancers. A critical factor for RT failure (the locoregional relapse or distant metastases) was that tumor-specific radioresistance^172^. Numerous studies demonstrated that tumor-driven exosomal circRNAs were closely associated with radioresistance. In hypopharyngeal squamous cell carcinoma, exosomal circCUX1 could induce tolerance to radiotherapy via regulating the expression of Caspase1 and decreasing the release of inflammatory factors 109. As an oncogene, circMYC in exosomes could enhance the radiotherapy resistance in nasopharyngeal carcinoma cells 110. A similar mechanism had been found in colon cancer. For instance, exosomal circ_IFT80 suppresses radiosensitivity in colorectal cancer by regulating miR-296-5p/MSI1 Axis 45 (Table 2).

### Tumor-driven exosomal circRNAs and immune infiltration in TME

**Figure 4:**
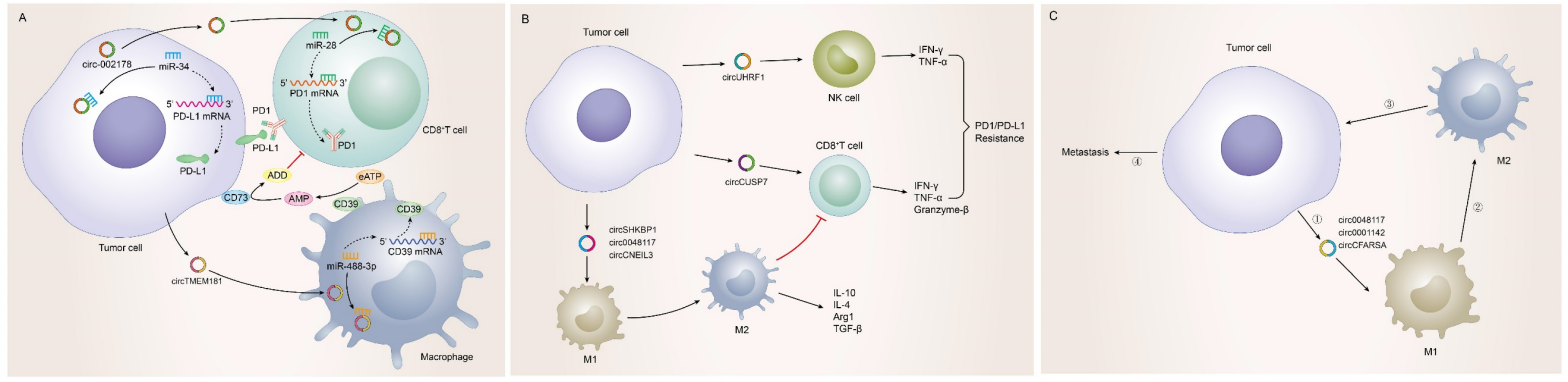
Tumor-driven exosomal circRNAs and immune infiltration in TME: **A**: Tumor cells regulate CD8+T cells and macrophages to participate in immune escape in TME by means of tumor-driven exosomal circRNAs; **B**: Tumor cells are involved in regulating PD1/PD-L1 drug resistance by regulating the secretion of cytokines by immune cells through exosome circRNAs; **C**: Tumor cells regulate immune cell-related tumor invasion and metastasis by means of tumor-driven exosomal circRNAs.

Possessing the capability of facilitating to interplay between cancer cells and surrounding cells, tumor-driven exosomal circRNAs had become a crucial role in intercellular communication 6, 9. The TME was exactly a complicated ecosystem composed of stromal cells, extracellular matrix (ECM) components and exosomes which ensured to orchestrate cell-cell interactions. As an important component of stromal cells, immune cells could recognize antigens from tumor cells to kill them. However, most cancers could evade immune system-mediated destruction due to the mutations of immune and apoptotic pathway genes or the dysregulation of epigenetic regulation of immune escape genes 173-175. circRNAs were closely related to the non-mutational epigenetic events in tumor immune escape. Numerous studies had identified many tumor-driven exosomal circRNAs epigenetically regulating immune infiltration, which mediated tumor immune escape and affected the efficacy of tumor immunotherapy. The specific mechanisms by which these circRNAs mediate immune cell infiltration and promote cancer development are summarized as follows: **I)** Immune escape: A research from HCC indicated that those HCC patients responding poorly to anti-PD1 therapy had high exosomal expression of circTMEM181. It was because of these high levels of exosomal circTMEM181 favored the immunosuppressive microenvironment. Exosomal circTMEM181 sponged miR-488-3p and upregulated CD39 expression in macrophages. Cell-specific CD39 expression in macrophages and CD73 expression in HCC cells synergistically activated the eATP-adenosine pathway which produces more adenosine, thereby impairing CD8+ T cell function and driving anti-PD1 resistance 27. In LUAD, exosomal has-circRNA-002178 was reported to be related to immune escape and acted dual roles. Firstly, circRNA-002178 could act as a sponge for miR-34a in tumor cells to promote the PDL1 expression in tumor cells. Then tumor-derived exosomes could deliver circRNA-002178 into CD8+ T cells which enhanced PD1 expression by sponging the miR-28-5p. The cumulative expression of PDL1 and PD1 in tumor cells and CD8+ T cells finally inhibited the activation of immune cells and helped cancer cells escape T-cell-mediated death and resistanti-tumor immune responses 84 (Figure 4A). **II)** Regulate the secretion of cytokines by immune cells: Cytokines were major regulators of innate and adaptive immunity that enable cells of the immune system to communicate over short distances managing the function of immune cells 176. For instance, Zhang et al. found that exosomal circUHRF1 promoted NK cell dysfunction in patients with HCC and inhibited NK cell-derived IFN-γ and TNF-α secretion thus driving resistance to anti-PD1 immunotherapy 19. In LUAD, exosomal circUSP7 inhibits CD8+ T cell secretion of cytokines, including TNF-α, IFN-γ, Granzyme-B and perforin to promote resistance to anti-PD1 immunotherapy in NSCLC patients 90. Similarly, exosomal circSHKBP1 could promote M2 polarization and increased the level of IL-10 and IL-4, simultaneously decreasing the secretion of TNF-a, IFN-g, granzyme-B, and perforin from CD8+ T cells 88. In EC, hsa-circ-0048117 could facilitate the M2 macrophage polarization and increase the secretions of Arg1, IL-10 and TGF-β 66. The changes in the levels of these cytokines ultimately affected the immune function of immune cells in the TME (Figure 4B). **III)** Immunosuppression and immune cell-mediated invasion and metastasis: Immunosuppression played an important role in TME to promote immune escape of tumor cells, which was the premise of promoting tumor development, metastasis and recurrence of nearby tumors, and resistance to therapy 177. Some reports also indicated that exosomal circRNAs were closely related to the immunosuppression in TME: The study from Yang et al. indicated that exosomal circPSMA1 functioned as a tumor promoter through the circPSMA1/miR-637/Akt1-β-catenin (cyclin D1) regulatory axis, which can facilitate the immunosuppression of triple-negative breast cancer 101. In glioma, exosomal circNEIL3 derived M2 macrophages infiltrated into the TME enabling them to acquire immunosuppressive properties by stabilizing IGF2BP3 and in turn promoting glioma progression 73. Infiltrating immune cells in tumor immune microenvironment had been reported to participate in tumor invasion and metastasis 178, 179. In this review, we also found that some tumor-derived exosomes were involved in the management of malignant tumor metastasis by affecting some immune cells. For instance, exosomal circ_0001142 in TME promoted macrophage polarization in BC in the presence of ERs, thus promoting the proliferation and metastasis of tumor cells 103. In NSCLC, tumor-derived exosomal circFARSA polarized the macrophages to a M2 phenotype via PTEN ubiquitination and degradation, which further activated the PI3K/AKT signaling pathway and induced tumor cell metastasis 89. In EC, exosomal hsa-circ-0048117 could act as sponge of miR-140 by competing with TLR4 to facilitate the M2 macrophage polarization eventually enhancing the ability of invasion and migration of tumor cells 66 (Figure 4C).

Of course, due to the complexity of the components in the TME, the 'cross-talk' interaction between tumor and stroma had a complex influence on the process of tumorigenesis and progression.

## Conclusions and perspectives

With the in-depth exploration of the pathogenesis in the field of cancer treatment, circRNAs carried by tumor exosomes gradually showed an important role in cancer. These circRNAs in tumor-driven exosomes can deliver tumor-specific circRNAs to corresponding sites to promote or inhibit carcinogenesis through adjacent (such as paracrine or autocrine secretion of tumor cells) or distant secretion. Under the regulation of various mechanisms, tumor-driven exosomal circRNAs regulated the proliferation, invasion, migration and chemo-or-radiotherapy sensitivity of tumor cells. It was worth noting that the expression of many tumor-driven exosomal circRNAs were also regulated by upstream factors including some RNA binding proteins, oligonucleotides and other modification enzymes, which to some extent increased the complexity and uncertainty of exosomal circRNAs involvement in tumorigenesis and development. Encouragingly, some exosomal circRNAs had shown clinical application values in the future, such as early diagnosis of cancer or indicating poor prognosis.

Although it is still difficult to solve a series problems of tumor development by interfering with single exosomal circRNA, with the further study of the role of exosomal circRNAs in tumor, we also found that there were still several aspects worth looking forward to in the future. Firstly, more studies on the formation mechanism of exosomal circRNAs may provide more ideas and options for researchers in terms of inhibiting tumor formation by blocking the secretion of exosomes carrying oncogene circRNAs in the future. In addition, with the unique transport function of exosomes, some circRNAs acting as tumor suppressor genes can exert tumor suppressor effects on tumor cells. For instance, exosomes carrying therapeutic functions had been studied in clinical trials over the past few years and it was expected to become a new model of cancer treatment under the supervision of strict operational standards 8. All the above mechanisms provide a theoretical basis for exosomal circRNAs as cancer therapeutic targets. Although the abundance of exosomal circRNAs is very low, exosomal circRNAs are widely present in human tissues and body fluids. Therefore, the research on exosomal circRNAs is a hot topic in the field of cancer diagnosis, efficacy or prognosis evaluation, and targeted therapy in future.

## Supplementary Material

Supplementary tables.

## Figures and Tables

**Table 1:** ceRNA network mediated by tumor-driven exosomal circRNAs in cancers

Exosomal circRNA			CircBase ID	miRNA	Target gene	Function	Ref.
Digestive system malignancy							
	Liver cancer						
		circTMEM45A	hsa_circ_0066659	miR-665	IGF2	Proliferation, metastasis	18
		circUHRF1	hsa_circ_0048677	miR-449c-5p	TIM-3	Natural killer cell exhaustion, drug resistance	19
		hsa_circ_0004658	hsa_circ_0004658	miR-499b-5p	JAM3	Proliferation, metastasis	20
		circ-DB	hsa_circ_0025129	miR-34a	USP7	Proliferation, deubiquitination	21
		circRNA Cdr1	hsa_circ_0001946	miR-1270	AFP	Proliferation, metastasis	25
		circ-G004213	hsa-circ-G004213	miR-513b-5p	PRPF39	Upregulating drug sensitivity	26
		circTMEM181	hsa_circ_0001663	miR-488-3p	CD39	Immunosuppression, drug resistance	27
		circ-0072088	hsa_circ_103809	miR-375	MMP-16	Metastasis	28
		circFBLIM1	hsa_circ_0010090	miR-338	LRP6	Glycolysis, metastasis, apoptosis	29
		circANTXR1	hsa_circ_0055033	miR-532-5p	XRCC5	Proliferation, metastasis	31
		circRNA_100284	hsa_circ_100284	miR-217	EZH2	Proliferation, metastasis	33
	Colon cancer						
		circ_0006174	hsa_circ_104852	miR-1205	CCND2	Proliferation, metastasis, drug resistance	37
		circFNDC3B	hsa_circ_006156	miR-937-5p	TIMP3	Metastasis, angiogenesis	38
		circ-133	has_circ_0010522	miR-133a	GEF-H1, RhoA	Proliferation, metastasis	39
		ciRS-122	hsa_circ_0005963	miR-122	PKM2	Glycolysis, drug resistance	41
		circPACRGL	has_circ_0069313	miR-142-3p	TGF-β1	Proliferation, metastasis, apoptosis	42
				miR-506-3p			
		circFMN2	hsa_circ_0005100	miR-1182	hTERT	Proliferation, metastasis	44
		circ_IFT80	hsa_circ_0067835	miR-296-5p	MSI1	Proliferation, metastasis, apoptosis, drug resistance	45
		circ-RNF121	hsa_circ_100876	miR-1224-5p	FOXM1	Glycolysis, proliferation, metastasis, apoptosis	46
		circLONP2	hsa_circ_0008558	miR-17	DGCR8	Metastasis	49
		circCOG2	hsa_circ_101555	miR-1305	TGF-β1	Proliferation, metastasis	50
		circ_0007334	hsa_circ_0007334	miR-577	KLF12	Proliferation, metastasis, apoptosis	53
	Gastric cancer						
		ciRS-133	hsa_circ_0010522	miR-133	PRDM16	Proliferation, metastasis, adipocyte metabolic	54
		circNEK9	hsa_circ_0032683	miR-409-3p	MAP7	Proliferation, metastasis	55
		circ-PVT1	circ-PVT1	miR-30a-5p	YAP1	Proliferation, metastasis, apoptosis, autophagy	56
		circ-RanGAP1	hsa_circ_0063526	miR-877-3p	VEGFA	Proliferation, metastasis	60
		circ_0000260	hsa_circ_0000260	miR-129-5p	MMP11	Proliferation, metastasis, apoptosis, drug resistance	61
		circ29	hsa_circ_0044366	miR-29a	VEGF	Proliferation, metastasis	62
		circNHSL1	hsa_circ_0006835	miR-149-5p	YWHAZ	Metastasis, glutaminolysis	63
	Bile duct/gallbladder cancer						
		circ_0020256	hsa_circ_0020256	miR-432-5p	E2F3	Proliferation, metastasis	64
	Esophagus cancer						
		circ0048117	hsa-circ-0048117	miR-140	TLR4	M2 macrophage polarization, metastasis	66
		circ-SFMBT2	hsa_circ_0000211	miR-107	SLC1A5	Proliferation, metastasis, glutaminolysis	67
	Pancreatic cancer						
		circZNF91	hsa-circ-0109315	miR-23b-3p	SIRT1	Glycolysis, drug resistance	68
		circ-PDE8A	hsa_circ_0036627	miR-338	MACC1	Metastasis	69
Nervous system malignancy							
	Glioma						
		circMMP1	hsa_circ_0024108	miR-433	HMGB3	Proliferation, metastasis, apoptosis	70
		circRNA 0001445	hsa_circ_0001445	miR-127-5p	SNX5	Proliferation, metastasis, apoptosis	75
Respiratory neoplasms							
	Lung cancer						
		circSATB2	hsa_circ_0008928	miR-326	FSCN1	Proliferation, metastasis	78
		circ_100395	hsa_circ_100395	miR-141-3p	LATS2	Proliferation, metastasis, regulating signaling pathway	79
		circ_102481	hsa_circRNA_102481	miR-30a-5p	ROR1	Metastasis, apoptosis, drug resistance	80
		circ_0076305	hsa_circ_0076305	miR-186-5p	ABCC1	Drug resistance	81
		circFBXW8	circFBXW8	miR-370-3p	TRIM44	Proliferation, metastasis	82
		circRNA-002178	has_circ_002178	miR-34	PD1/PDL1	Immunotherapy, diagnosis	84
		circ_PIP5K1A	hsa_circ_0014130	miR-101	ABCC1	Proliferation, metastasis, apoptosis, drug resistance	85
		hsa_circ_0002130	hsa_circ_0002130	miR-498	GLUT1/HK2/LDHA	Glycolysis, drug resistance	86
		circSHKBP1	hsa_circ_0000936	miR-1294	PKM2	M2 macrophage polarization, proliferation, metastasis	88
		circUSP7	hsa_circ_0005152	miR-934	SHP2	Immune infiltration, drug resistance	90
	Malignant pleural mesothelioma						
		circPLK1	hsa_circ_0038632	miR-1294	HMGA1	Proliferation, metastasis	92
Female malignant tumor							
	Ovarian cancer						
		circWHSC1	hsa_circ_0001387	miR-145	MUC1	Proliferation, metastasis	93
				miR-1182	hTERT		
		circ-0001068	hsa_circ_0001068	miR-28-5p	PD1	Immunotherapy	95
		circFoxp1	hsa_circ_0001320	miR-22	CEBPG	Proliferation, apoptosis, drug resistance	97
				miR-150-3p	FMNL3		
		circPUM1	circPUM1	miR-615-5p	NF-kB	Proliferation, metastasis, apoptosis	98
				miR-6753-5p	MMP2		
	Breast cancer						
		circHIF1A	hsa_circ_0032138	miR-580-5p	CD44	Proliferation	99
		circPSMA1	circPSMA1	miR-637	Akt1	Immune infiltration, proliferation, metastasis	101
		circ-MMP11	hsa_circ_0062558	miR-153-3p	ANLN	Proliferation, metastasis, apoptosis, drug resistance	102
		circ_0001142	hsa_circ_0001142	miR-361-3p	PIK3CB	M2 macrophage polarization, proliferation, metastasis	103
		circCARM1	hsa_circ_0004552	miR-1252-5p	PFKFB2	Glycolysis	104
Head and neck malignancy							
	Oral squamous cell carcinoma						
		circGDI2	hsa_circ_0005379	miR-424-5p	SCAI	Glycolysis, proliferation, metastasis	106
		circSPATA6	hsa_circ_0008202	miR-182	TRAF6	Proliferation, metastasis, apoptosis	108
	Laryngeal squamous cell carcinoma						
		circRASSF2	hsa_circ_0059354	miR-302b-3p	IGF-1R	Proliferation, apoptosis	111
Urinary malignancy							
	Bladder carcinoma						
		circPRMT5	hsa_circ_101320	miR-30c	SNAIL1	Metastasis	119
	Prostate cancer						
		circXIAP	hsa_circ_0005276	miR-1182	TPD52	Proliferation, metastasis, apoptosis, drug resistance	120
		circRNA HIPK3	hsa_circ_0000284	miR-212	BMI-1	Proliferation, metastasis, apoptosis	122

**Table 2:** Tumor-driven exosomal circRNAs and clinical applications

Clinical objective		Tumor-driven exosomal circRNAs
Diagnosis	Cancer diagnosis and early cancer screening	circLPAR1, circ-PNN, circ_0055202, circ_0074920, circ_0043722, circRNA-002178, circKIAA1244, circ_0004001, circ_0004123, circ_0075792, circ_0065149, circ-0004771, circ_0003828, circ_0075828, circ_0002976, circ_0069313
	Predict cancer recurrence and metastasis	circ_30029, circ_117300, circ_176436, circ_112897, circ_112897, circ_178252, circ_115617, circ_14736, circ_17720, circ_0000199, circMYC, circ_0005019, circ_0000880, circ_0051680, circ_0006365, circ_0056616, circ_0019201, circ_0011773, circ_0122790, circ_0006602
	Predicting treatment sensitivity	circMYC
Treatment	Promote chemotherapy resistance	circ_0058493, circ_0006174, circUHRF1, circZNF91, circ_102481, circRNA-SORE, ciRS-122, circ_0042003, circ_PIP5K1A, circ_103801, circ_0002130, circFoxp1, circMYC, circTMEM181, circ_0000338, circUSP7
	Promote radiation resistance	circCUX1, circMYC, circ_IFT80
	Increasing chemotherapy sensitivity	Cdr1, circ_G004213
