# Supplementary material for: Regulatory mechanisms and clinical applications of tumor-driven exosomal circRNAs in cancers
Source: Int J Med Sci. 2023 May 8;20(6):818–35. doi: 10.7150/ijms.82419 (PMC10198146; doi:10.7150/ijms.82419)
Supplement: Supplementary file 1 — Supplementary tables. [file ijmsv20p0818s1.pdf]

Supplementary Table1 Basic informations of tumor-driven exosomal circRNAs in cancers

	Exosomal circRNA	Source of exosome	Receive cell	Expression	Ref
Digestive system malignancy					
Liver cancer					
	hsa_circ_0004277	Serum of Patients,HepG2,SMMC-7721	HL-7702	Upregulated	17
	circTMEM45A	Serum of Patients	—	Upregulated	18
	circUHRF1	Serum of Patients,HCCLM3,SMMC-7721	NK	Upregulated	19
	hsa_circ_0004658	THP-1 Mφ	SMMC-7721,HepG2	Upregulated	20
	hsa_circ_0025129	Serum of Patients,3T3L1	HepG2	Upregulated	21
	circRNA-SORE	Drug resistant HCC cells-derived Exosomes	parental HCC cell	Upregulated	22
	circPTGR1	Serum of Patients,HCCLM3	HepG2,97L	Upregulated	23
	hsa_circ_0004001,hsa_circ_0004123,hsa_circ_0075792	Serum of Patients	—	Upregulated	24
	circRNA Cdr1	Serum of Patients,HepG2,SMMC-7721	293T	Upregulated	25
	circ-G004213	Serum of Patients	—	Upregulated	26
	circTMEM181	Serum of Patients<br>Huh-7 overexpressing circTMEM181	tumor-infiltrating immune cells	Downregulated	27
	circ-0072088	Serum of Patients,Huh-7	Hep3B	Upregulated	28
	circFBLIM1	Serum of Patients	—	Upregulated	29
	circRNA-100338	Serum of Patients,Hep3B,MHCC97H	HUVEC	Upregulated	30
	circANTXR1	Serum of Patients,Huh-7	HCCLM3	Upregulated	31
	circ_0006602	Serum of Patients	—	Upregulated	32
	circRNA_100284	Serum of Patients,Arsenite-transformed L-02	No-transformed L-02	Upregulated	33
	circ_0051443	Serum of Patients,HL-7702	Hep3b,HuH7	Downregulated	34
Colon cancer					
	circLPAR1	Serum of Patients,FHC,HCT116,DLD1	HCT116,DLD1	Upregulated	35
	circ-ABCC1	Serum of Patients,CD133+Caco2	Caco2,HCT15	Upregulated	36
	circ_0006174	DOX-LoVo,DOX-HCT116	LoVo,HCT116	Upregulated	37
	circFNDC3B	Serum of Patients,LoVo,SW480	LoVo,SW480	Downregulated	38
	circ-133	Serum of Patients<br>hypoxic-HCT116,hypoxic-SW480	HCT116,SW480	Upregulated	39
	circ-PNN	Serum of Patients	—	Upregulated	40
	ciRS-122	Serum of Patients,SW480,SW480/L-OHP	SW480	Upregulated	41
	circPACRGL	HCT116,SW480	HCT116,SW480	Upregulated	42
	hsa-circ-0004771	Serum of Patients	—	Upregulated	43
	circFMN2	Serum of Patients	—	Upregulated	44

	circ_IFT80	Serum of Patients	SW480,SW620	Upregulated	45
	circ-RNF121	HCT-116,SW480	—	Upregulated	46
	circEPB41L2	Serum of Patients,FHC,SW480,SW620	SW480,SW620	Downregulated	47
	circ_0000338	HCT116-R	HCT116-P	Upregulated	48
	circLONP2	HCT116-HM	HCT116	Upregulated	49
	circCOG2	HCT8-HM,SW480-HM	HCT8-LM,SW480-LM	Upregulated	50
	circRNAs	DLD-1,DKO-1,DKs-8	—	—	51
	circRHOBTB3	Serum of Patients	—	Downregulated	52
	circ_0007334	FHC,HCT-116,SW480		Upregulated	53
Gastric cancer					
	hsa_circ_0010522	Serum of Patients,SGC7901	3T3L1	Upregulated	54
	hsa_circ_0032683	Serum of Patients	XGC-1	Upregulated	55
	circ-PVT1	Serum of Patients	—	Upregulated	56
		HGC-27/DDP,AGS/DDP			
	circ-KIAA1244	Serum of Patients	—	Downregulated	57
	hsa_circ_0065149	Serum of Patients	—	Upregulated	58
	circRNAs	Serum of Patients	—	—	59
	circ-RanGAP1	Serum of Patients	—	Upregulated	60
	circ_0000260	Serum of Patients	—	Upregulated	61
	circ_0044366	Serum of Patients	HUVECs	Upregulated	62
		ov-circ29/si-circ29-SGC-7901			
	circNHSL1	Serum of Patients,HGC-27,AGS	—	Upregulated	63
Bile Duct/gallbladder Cancer					
	circ_0020256	M2 macrophages,	HCCC-9810,RBE	Upregulated	64
	circRNAs	Serum of Patients	—	—	65
Esophagus cancer					
	hsa-circ-0048117	Serum of Patients,Eca-109	M2 macrophage	Upregulated	66
	circ-SFMBT2	KYSE150,TE1	GW4869	Upregulated	67
Pancreatic cancer					
	circZNF91	hypoxia-BxPC-3,hypoxia-SW1990	BxPC-3,SW1990	Upregulated	68
	circ-PDE8A	Hs 766T,Hs 766T-L2	BxPC-3,Capan-1	Upregulated	69
Nervous system malignancy					
Glioma					
	circMMP1	Serum of Patients	—	Upregulated	70
	circRNAs	Serum of Patients	—	—	71
	hsa_circ_0042003	Serum of Patients	—	Upregulated	72

	circNEIL3	Serum of Patients,U251,A172	U251,A172	Upregulated	73
	hsa_circ_0002874	U87,U251	—	Upregulated	74
	circRNA 0001445	A172,U373	A172,U373	Upregulated	75
Astrocytoma					
	circRNAs	Serum of Patients	—	—	76
	Respiratory neoplasms				
Lung cancer					
	circRNA_101093	Serum of Patients,A549,H1299	A549,H1299	Upregulated	77
	circSATB2	H1299	BEAS-2B,H460,A549	Upregulated	78
	circ_100395	adipose-derived mesenchymal stem cell	H1650	Downregulated	79
	circRNA_102481	Serum of Patients,PC9/GR,HCC827/ER	PC9/GR,HCC827/ER	Upregulated	80
	circ_0076305	Serum of Patients	A549,H292	Upregulated	81
	circFBXW8	A549,PC9	—	Upregulated	82
	has-circRNA-002178	Serum of Patients,PC9	CD8+T cells	Upregulated	84
	circ_PIP5K1A	Serum of Patients,A549,H1299	A549,H1299	Upregulated	85
	hsa_circ_0002130	Serum of Patients	—	Upregulated	86
	hsa_circRNA_0056616	Serum of Patients,PC9	Serum of Patients	Upregulated	87
	hsa_circ_0069313	Serum of Patients	—	Upregulated	83
	circSHKBP1	Serum of Patients,A549,H1299	A549,H1299	Upregulated	88
	circFARSA	A549,PC9	macrophages,A549,PC9	Upregulated	89
	circUSP7	Serum of Patients,A549,H460	CD8+T cells	Upregulated	90
	circRNAs	Serum of Patients	—	—	91
Malignant Pleural Mesothelioma					
	circPLK1	Serum of Patients	—	Upregulated	92
	Female malignant tumor				
Ovarian cancer					
	circWHSC1	ov-circWHSC1-HMrSV5	CAOV3,OVCAR3	Upregulated	93
	circCdr1	Serum of cisplatin_resistant ovarian patients	—	Downregulated	94
	circ-0001068	Serum of Patients,IOSE80,A2780	Jurkat T cells	Upregulated	95
	circRNAs	Serum of Patients	—	—	96
	circFoxp1	Serum of Patients	COC1,SKOV3	Upregulated	97
	circPUM1	ov-circPUM1-CAOV3	HMrSV5	Upregulated	98
Breast cancer					
	circ_0032138	Serum of Patients	MDA-MB-231,SKBR3	Upregulated	99
		hypoxic CAFs,CAFs-circRNA control/N-Exo			

	circRNA-0005795,circRNA-0088088	Serum of Patients	—	Upregulated/Downregulated	100
	circPSMA1	Serum of Patients,MDA-MB-231,BT-549	MDA-MB-231	Upregulated	101
	circ-MMP11	Serum of Patients<br>lapatinib resistance MDA-MB-231,MCF-7		Upregulated	102
	circ_0001142	MB231,MB453	THP-1,BC	Upregulated	103
	circCARM1	MDA-231	MDA-231	Upregulated	104
	circHIF1A	ov-circHIF1A/pLCDH-MDA-231	MDA-231	Upregulated	105
Head and neck malignancy					
Oral Squamous Cell Carcinoma					
	circGDI2	CAL-27,SCC-15， ov-circGDI2-Donor	CAL-27,SCC-15	Downregulated	106
	circ_0000199	Serum of Patients	—	Upregulated	107
	circSPATA6	ov-circSPATA6-CAL-27/HSC6	CAL-27,HSC6	Downregulated	108
Nasopharyngeal carcinoma					
	circCUX1	Serum of Patients	—	Upregulated	109
	circMYC	Serum of Patients	—	Upregulated	110
Laryngeal squamous cell carcinoma					
	circRASSF2	Serum of Patients	—	Upregulated	111
	circRNAs	Serum of Patients	—	—	112
Thyroid Carcinoma					
	circRNAs	Serum of Patients	—	—	113
Hematological malignancy					
Multiple myeloma					
	circRNAs	Serum of Patients	—	—	114
	circ-ATP10A	Serum of Patients	—	Upregulated	115
	circMYC	Serum of Patients	—	Upregulated	116
Chronic Lymphocytic Leukemia					
	circ_0058493	K562,K562/G01	—	Upregulated	117
	circMC-COX2	Serum of Patients	—	Upregulated	118
Urinary malignancy					
Bladder Carcinoma					
	circPRMT5	Serum of Patients	—	Upregulated	119
Prostate Cancer					
	circXIAP	Docetaxel-resistance-DU145/PC3	—	Upregulated	120
	circRNAs	—	—	—	121
	circRNA HIPK3	Serum of Patients	22Rv1,DU145	Upregulated	122

	Exo-si/NC-circHIPK-3-22Rv1,DU145			
circ_0044516	Serum of Patients,PC3,2B4,2RV1	—	Upregulated	123
Osteosarcoma				
circ_103801	Serum of Patients,MG63,MG63/CDDP	MG63	Upregulated	124
circRNAs	HUVEC	—	—	125

SupplementaryTable 2 Abbreviation and interpretation in this review

Abbreviation and interpretation
AA:arachidonic acid
ABCC1:ATP binding cassette subfamily C member 1
ABCG2:ATP binding cassette subfamily G member 2
ATP:Adenosine triphosphate
BAT:brown adipose tissue
BC:breast cancer
CCND2:cyclin D2
CDDP:cisplatin
CEA:carcinoembryonic antigen
CEBPG:CCAAT enhancer binding protein gamma
ceRNA:competing endogenous RNAs
CML:chronic myelocytic leukemia
CRC:colorectal cancer
EC:esophageal cancer
ECM:extracellular matrix
eIF4A3:eukaryotic translation initiation factor 4A3
EMT:epithelial-to-mesenchymal transition
ENO1:enolase 1
ENO2:enolase 2
ER:estrogen receptor
EWSR1 :EWS RNA binding protein 1
FABP3:fatty acid binding protein 3
FAS:fatty acid synthesis
FAs:fatty acids
FMNL3:formin like 3
FOXM1:Forkhead box M1
FUS:FUS RNA binding protein

GBM:glioblastoma multiforme
GC:gastric cancer
GEM:gemcitabine
GLUTs:glucose transporters
HCC:hepatocellular carcinoma
HECTD4:HECT domain E3 ubiquitin protein ligase 4
HIF-1α:hypoxia inducible factor 1 subunit alpha
HK:hexokinase
HUVECs:human umbilical vein endothelial cells
IGF2BP:insulin-like growth factor 2 mRNA-binding protein
LDHA:lactate dehydrogenase
LRP6:LDL receptor related protein 6
LUAD:lung adenocarcinoma
M1:M1 subphenotype macrophage
M2:M2 subphenotype macrophage
METTL3:methyltransferase 3
MM:multiple myeloma
MMP:matrix metalloproteinase
MSI1:musashi RNA binding protein 1
NANOG:Nanog homeobox
NcRNAs:non-coding RNAs
NEIL3:nei like DNA glycosylase 3
NFIB:nuclear factor IB
NLCSC:non-small cell lung cancer
OC:ovarian cancer
OCT4:also known as POU5F1:POU class 5 homeobox 1
OSCC:squamous cell carcinoma
OXPHOS:oxidative phosphorylation
PC:pancreatic carcinoma
PCA:pancreatic cancer
PCa:prostatic cancer
PD1:programmed cell death protein 1
PD-L1:programmed cell death 1 ligand 1
PFK:6-phosphfructa-1-kinase
PFKFB2:6-phosphofructo-2-kinase/fructose-2,6-biphosphatase 2
PKM:pyruvate kinase

PRDM16:PR/SET domain 16
PRP19:pre-mRNA processing factor 19
PTEN:phosphatase and tensin homolog
RBP:RNA-binding proteins
ROS:reactive oxygen species
RT:radiation therapy
SCAI:suppressor of cancer cell invasion
SHP2:K-box region and MADS-box transcription factor family protein
SIRT1:sirtuin 1
SOX2:SRY-box transcription factor 2
TFs:transcription factors
TIM-3:also known as HAVCR2:hepatitis A virus cellular receptor 2
TLR4:toll like receptor 4
TME:tumor microenvironment
TMZ:temozolomide
USP7:ubiquitin specific peptidase 7
VEGFA:vascular endothelial growth factor A
WAT:white adipose tissue
YBX1:Y-box binding protein 1
